# Benign infantile convulsion as a diagnostic clue of paroxysmal kinesigenic dyskinesia: a case series

**DOI:** 10.1186/1752-1947-8-174

**Published:** 2014-06-01

**Authors:** Naoya Matsumoto, Satoru Takahashi, Akie Okayama, Akiko Araki, Hiroshi Azuma

**Affiliations:** 1 Department of Pediatrics, Asahikawa Medical University, 2-1-1-1 Midorigaoka-Higashi, Asahikawa, Hokkaido 078-8510, Japan

**Keywords:** Benign infantile convulsion, Mutation, Paroxysmal kinesigenic dyskinesia, *PRRT2*, Seizures

## Abstract

**Introduction:**

Paroxysmal kinesigenic dyskinesia is characterized by sudden attacks of involuntary movements. It is often misdiagnosed clinically as psychogenic illness, which distresses the patients to a great extent. A correct diagnosis will improve the quality of life in patients with paroxysmal kinesigenic dyskinesia because treatment with low doses of anticonvulsants is effective for eliminating the clinical manifestations. Paroxysmal kinesigenic dyskinesia can occur independently of or concurrently with benign infantile convulsion. Identification of *PRRT2* as the causative gene of benign infantile convulsion and paroxysmal kinesigenic dyskinesia allows genetic confirmation of the clinical diagnosis.

**Case presentation:**

We describe the clinical features of a Japanese family with either paroxysmal kinesigenic dyskinesia or benign infantile convulsion. A *PRRT2* missense mutation (c.981C > G, p.Ile327Met) was identified in two patients with benign infantile convulsion and three patients with paroxysmal kinesigenic dyskinesia as well as in two unaffected individuals. Allowing incomplete penetrance in the mutation carriers, this mutation co-segregated completely with the phenotype. The patients with paroxysmal kinesigenic dyskinesia had been misdiagnosed with psychogenic illness for many years. They were correctly diagnosed with paroxysmal kinesigenic dyskinesia when their children visited a pediatrician for benign infantile convulsion. Treatment with carbamazepine controlled their involuntary movements completely.

**Conclusions:**

Paroxysmal kinesigenic dyskinesia is a treatable movement disorder that is often misdiagnosed clinically as psychogenic illness. It is important to note that two clinically distinct disorders, benign infantile convulsion and paroxysmal kinesigenic dyskinesia, are allelic conditions caused by *PRRT2* mutations. Paroxysmal kinesigenic dyskinesia should be suspected in families with a child with benign infantile convulsion.

## Introduction

Paroxysmal dyskinesias are episodic movement disorders characterized by sudden attacks of involuntary movements, such as dystonia, choreoathetosis, and ballism. Most patients are neurologically normal between the attacks and remain conscious through these attacks. On the basis of the events that trigger the abnormal movements, paroxysmal dyskinesias are subdivided into paroxysmal kinesigenic dyskinesia (PKD), paroxysmal exercise-induced dyskinesia (PED), and paroxysmal non-kinesigenic dyskinesia (PNKD) [1,2]. In patients with PKD, the attacks of abnormal movements are triggered by sudden voluntary movements and last less than 1 minute [3]. The attacks from PED are caused by prolonged exercise and disappear after cessation of physical exercise [4]. PED attacks last between 5 to 30 minutes. By contrast, in patients with PNKD, the attacks occur spontaneously but may be exacerbated by alcohol or caffeine consumption, emotional stress, and fatigue [5]. PNKD attacks last minutes to hours and are typically longer than PKD attacks. Most cases of PKD are familial and inherited in an autosomal dominant trait.

*PRRT2* on chromosome 16 (16p11.2), which encodes the Proline-rich transmembrane protein 2 (PRRT2), has been identified as the cause of the disease, although its precise role in PKD remains unclear [6]. PKD can occur independently of or concurrently with benign infantile convulsion (BIC) that is characterized by nonfebrile convulsions with onset between 3 and 12 months of age and favorable outcome with normal psychomotor development [7]. Typical seizures are focal with or without secondary generalization and usually occur in clusters.

The diagnosis of PKD is based on the clinical features and the correct diagnosis has implications for treatment and prognosis. However, PKD is often misdiagnosed clinically as epilepsy or psychogenic illness. Recent genetic discoveries will increase the chances of a correct diagnosis and contribute to elucidating the pathophysiology of this disease. Here we report the clinical features of familial cases with either BIC or PKD. The *PRRT2* mutation identified in the present study has been reported in a patient with BIC without PKD [8].

## Case presentation

**Figure 1:**
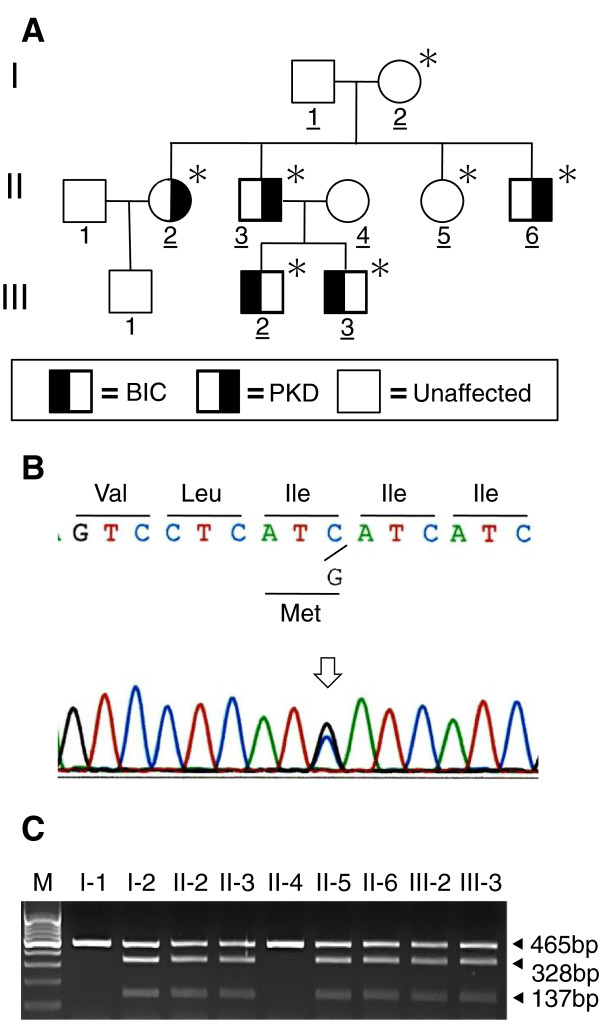
**A familial case with benign infantile convulsion and paroxysmal kinesigenic dyskinesia and *PRRT2 *mutation. A**: Affection status for benign infantile convulsion and paroxysmal kinesigenic dyskinesia are as noted. Underlined pedigree numbers denote individuals whose deoxyribonucleic acid (DNA) was available and who were analyzed in the present study. Asterisks denote *PRRT2* mutation carriers. **B**: Automated DNA sequencing using the polymerase chain reaction product from the patients with either benign infantile convulsion or paroxysmal kinesigenic dyskinesia showed a C-to-G transition at nucleotide 981 in exon 3 of *PRRT2* (NCBI accession NM_145239.2), as indicated by the arrow, which resulted in an isoleucine-to-methionine substitution at amino acid position 327 (p.Ile327Met). **C**. BclI digestion of the exon 3 polymerase chain reaction product showed additional fragments (328bp and 137bp) in two members with benign infantile convulsion (III-2 and III-3) and three members with paroxysmal kinesigenic dyskinesia (II-2, II-3, and II-6) as well as in two unaffected members (I-2 and II-5), which resulted from a C-to-G transition creating a new BclI restriction site. These additional fragments were observed with the wild-type fragment (465bp), confirming the heterozygous mutation. Abbreviations: BIC, benign infantile convulsion; paroxysmal kinesigenic dyskinesia, PKD; bp, base pairs; Val, valine; Leu, leucine; Ile, isoleucine; Met, methionine.

Nine individuals of a Japanese family spanning three generations participated in the study. A detailed family history was obtained by interviewing each family member. Five members had either BIC (Patients 1 and 2, as described below) or PKD (Patients 3, 4, and 5) (Figure 1A). No participant had both PKD and BIC.

Patient 1 (III-2): a boy, now aged 7 years, experienced three afebrile seizures within 1 week at the age of 7 months. The seizures were characterized by a loss of consciousness and hypotonia and lasted from 1 to 3 minutes. His growth and developmental milestones were normal. Interictal electroencephalography (EEG) and cranial computed tomography (CT) revealed normal findings. He was treated with carbamazepine until 2 years of age, and the seizures disappeared and did not reoccur after cessation of the treatment. He has not shown any abnormal paroxysmal movements so far.

Patient 2 (III-3): a boy, now aged 5 years and the younger brother of Patient 1, presented with generalized tonic–clonic seizures that occurred in clusters at 7 months of age. He had normal neurological development. Interictal EEG and cranial CT findings were normal. He was started on carbamazepine and achieved complete seizure control. After cessation of treatment at 2 years of age, he has experienced neither seizure recurrences nor paroxysmal dyskinesias.

Patient 3 (II-2): a woman who had no history of infantile convulsions. At 12 years of age, she presented with abnormal paroxysmal movements occurring several times a day. These episodes were triggered by sudden voluntary movement, such as standing up from a chair, or by emotional stress, for example, when she was asked to write on the blackboard at school. The involuntary movements consisted of choreoathetosis and dystonia and lasted less than a minute in duration. She remained conscious during the episodes of involuntary movements. Unfortunately, she was misdiagnosed as having psychogenic illness. She had been distressed for many years until a physician made a correct diagnosis of PKD at 25 years of age. Treatment with carbamazepine controlled her involuntary movements completely.

Patient 4 (II-3): the father of the patients with BIC (III-2 and III-3) had no seizures in his infancy. He displayed intermittent involuntary movements from the age of 10 years. The nature of the movement was dystonic. The attacks were usually triggered by sudden movements or intention to move; for example, when he was going to take an elevator. The severity of attacks varied from episodes severe enough to cause him to fall down to mild episodes that were barely noticed by his friends and relatives. The attacks were never associated with an alteration in his level of consciousness. He had been bothered with involuntary movements for many years. Finally, he was diagnosed with PKD when his child (III-3) visited a pediatrician for BIC. His symptoms disappeared following the start of treatment with carbamazepine.

Patient 5 (II-6): an intellectually disabled man, now aged 24 years, who had a history of perinatal asphyxia and surgically repaired tetralogy of Fallot. He presented with generalized tonic–clonic seizures at 6 years of age. Although a detailed clinical description of his seizures was not available, his seizures were under control with phenobarbital and phenytoin. Family members have occasionally witnessed his dystonic attacks when he forgot to take medicine. The attacks seemed to be provoked by sudden voluntary movements and occurred independently of his habitual seizures. He remained conscious throughout the dystonic attacks.

After obtaining written informed consent from the participants, genomic deoxyribonucleic acid (DNA) was extracted from the peripheral blood leukocytes of each participant and used as the template for polymerase chain reaction (PCR). The compatible primers were used to yield DNA fragments spanning the entire coding region and intron–exon boundaries of *PRRT2*. The PCR fragments were analyzed using automated sequencing. The mutation identified by direct DNA sequencing was further confirmed by restriction fragment length polymorphism analysis. We identified a *PRRT2* missense mutation (c.981C > G, p.Ile327Met) in two participants with BIC and three participants with PKD in this family but also in two unaffected members (Figure 1B and 1C).

## Discussion

We report a familial case with a heterozygous mutation in *PRRT2* that has been identified as the causative gene of PKD. In this family, five individuals had either BIC or PKD. Although BIC and PKD can occur either alone or together, no individual had both BIC and PKD. The *PRRT2* missense mutation (c.981C > G, p.Ile327Met) was identified in two patients with BIC and three patients with PKD as well as in two unaffected individuals. The same *PRRT2* mutation has been identified in a patient with BIC without PKD [8]. Allowing incomplete penetrance in the mutation carriers, this mutation co-segregated completely with the phenotype. Our results confirmed that two clinically distinct disorders, BIC and PKD, might be allelic conditions caused by *PRRT2* mutation. Taking the temporal changes in the expression of the disease into consideration, we could not exclude the possibility that the younger patients with BIC, now aged 5 and 7 years, may develop dyskinesia later in life.

Attacks of PKD and epileptic seizures have several characteristics in common; both show paroxysmal motor manifestations and good response to anticonvulsants. On the basis of neurophysiological and imaging studies, a subcortical origin related to basal ganglia is proposed as being involved in the PKD pathogenesis [9-11], whereas BIC is relevant to cortical origin [12]. However, the co-occurrence of BIC and PKD in a family with a *PRRT2* mutation suggests that a subset of epileptic seizures may share a common pathogenic mechanism with PKD. PRRT2 is expressed in brain regions, including the cerebral cortex and basal ganglia, which were implicated in BIC and PKD, respectively [13]. PRRT2 has been shown to interact with the synaptosomal-associated protein of 25 kDa (SNAP25), a presynaptic membrane protein involved in the synaptic vesicle membrane docking and fusion pathway [14]. Thus, interaction of PRRT2 with SNAP25 may play a pivotal role in the regulation of neurotransmitter release [15]. Such a disturbance of synaptic function may be the common pathogenic mechanism underlying motor manifestations in BIC and PKD. The mechanism of age-dependent occurrence and remission of clinical manifestations in BIC and PKD remains to be elucidated.

## Conclusions

Identification of *PRRT2* as the causative gene of BIC and PKD allows genetic confirmation of the clinical diagnosis as well as genetic counseling to family members. PKD is often misdiagnosed clinically as epilepsy or psychogenic illness, which distresses the patients to a great extent. PKD should be suspected in families with a child with BIC. A correct diagnosis will improve the quality of life in patients with PKD because treatment with low doses of anticonvulsants, such as carbamazepine and phenytoin, is effective for eliminating the clinical manifestations.

## Consent

This study was approved by the Ethics Committee of Asahikawa Medical University (Approval number: 1043; Title: Elucidation of the molecular genetic basis of benign infantile convulsion and paroxysmal kinesigenic choreoathetosis). Written informed consent for the collection of peripheral blood samples and subsequent analyses was obtained from all participants, with the parents giving consent for themselves and on behalf of their children. Concurrently, written informed consent was obtained from the participants for publication of this case report and any accompanying images. A copy of the written consent is available for review by the Editor-in-Chief of this journal.

## Abbreviations

BIC: benign infantile convulsion; CT: computed tomography; EEG: electroencephalography; PCR: polymerase chain reaction; PED: paroxysmal exercise-induced dyskinesia; PKD: paroxysmal kinesigenic dyskinesia; PNKD: paroxysmal non-kinesigenic dyskinesia; PRRT2: Proline-rich transmembrane protein 2; SNAP25: synaptosomal-associated protein of 25 kDa.

## Competing interests

The authors declare that they have no competing interests.

## Authors’ contributions

NM carried out the molecular genetic studies and drafted the manuscript; ST identified the patients, carried out the clinical characterizations, and drafted the manuscript; AO and AA conceived the study and participated in its design; HA participated in the design of the study and helped to draft the manuscript; all authors read and approved the final manuscript.
